# Supplementary material for: Prevalence of Musculoskeletal Disorders and Their Associated Risk Factors among Furniture Manufacturing Workers in Guangdong, China: A Cross-Sectional Study
Source: Int J Environ Res Public Health. 2022 Nov 4;19(21):14435. doi: 10.3390/ijerph192114435 (PMC9654235; doi:10.3390/ijerph192114435)
Supplement: Supplementary file 1 [file ijerph-19-14435-s001.zip › ijerph-1913894-supplementary.pdf]

Table S1. Univariate analyses of risk factors for WMSDs occurring in neck and trunk regions among furniture manufacturing workers.

Factor	Neck			Upper back			Lower back			Hands/wrists			Elbows		
	n	**χ2**	***P***	***n***	**χ2**	***P***	***n***	**χ2**	***P***	***n***	**χ2**	***P***	***n***	**χ2**	***P***
Gender		3.68	0.05		0.55	0.45		1.56	0.21		3.35	0.06		0.92	0.33
Male	474			346			335			411			298		
Female	227			134			123			145			112		
Age (years)		7.63	0.05		5.06	0.16		0.80	0.84		2.96	0.39		2.72	0.43
<25	91			64			49			68			53		
25~	372			250			240			289			209		
35~	186			129			139			165			122		
45~	52			37			30			34			26		
BMI (Body mass index, kg/m^2)		5.13	0.16		1.15	0.76		3.16	0.36		7.34	0.06		6.08	0.10
<18.5	90			58			57			65			54		
18.5~	446			311			305			349			261		
24.0~	143			95			85			126			86		
28.0~	22			16			11			16			9		
Career length (years)		10.39	0.01*		12.34	<0.01*		1.04	0.79		0.66	0.88		3.23	0.35
1~2	460			321			315			398			283		
3~5	180			116			106			113			98		
6~10	40			22			25			32			18		
10~	21			21			12			13			11		
Educational level		18.18	<0.01*		8.06	0.04*		7.54	0.05		7.37	0.06		11.12	0.01*
Junior high school and below	381			273			276			348			256		
High school or technical secondary school	243			162			151			172			129		
College or university	74			42			29			34			22		
Postgraduate degree and above	b			b			b			b			b		
Marital status		0.94	0.62		0.67	0.71		1.94	0.37		0.01	0.99		0.59	0.74
Never married	228			160			143			190			134		
Married	450			308			302			349			265		
Else (divorced or widowed)	23			12			13			17			11		
Monthly income		1.89	0.59		3.10	0.37		10.07	0.01*		3.08	0.37		1.59	0.66
≤1000RMB	7			b			8			6			b		
1001-3000RMB	18			8			13			17			13		
3001-5000RMB	295			209			199			229			171		
＞5000RMB	381			258			238			304			222		
Physical exercise		8.89	0.06		3.37	0.49		4.65	0.32		3.83	0.42		5.00	0.28
Never	269			175			177			212			149		
Sometimes	368			258			239			290			225		
2~3 times a month	25			18			12			19			13		
1~2 times a week	28			17			18			21			13		
>3 times a week	11			12			12			14			10		
Smoking habits		0.15	0.69		5.43	0.02*		10.83	<0.01*		3.61	0.05		0.75	0.38
Non-smokers	372			234			213			278			212		
Smokers	329			246			245			278			198		

Physical health status		147.52	<0.01*		74.90	<0.01*		105.06	<0.01*		43.07	<0.01*		44.21	<0.01*
Good	263			193			163			247			173		
Moderate	363			237			245			262			200		
Poor	61			40			40			37			27		
Very poor	14			10			10			10			10		
Dominant hand		1.83	0.17		0.60	0.43		0.93	0.33		1.14	0.28		1.15	0.28
Right	647			432			411			499			367		
Left	54			48			47			57			43		
Type of work		0.12	0.72		4.43	0.03*		7.73	<0.01*		24.37	<0.01*		17.37	<0.01*
Frontline workers	610			434			419			522			385		
Other staff*	91			46			39			34			25		
Prolonged standing	645	0.03	0.85	442	0.04	0.83	428	1.77	0.18	529	9.32	<0.01*	383	1.49	0.22
Prolonged sitting	220	1.98	0.15	148	0.71	0.39	130	0.15	0.69	125	13.92	<0.01*	101	4.54	0.03*
Prolonged squatting /kneeling	252	13.28	<0.01*	189	21.73	<0.01*	184	24.36	<0.01*	219	25.78	<0.01*	164	20.78	<0.01*
Carrying heavy loads (more than 20 kg each time)	523	15.65	<0.01*	382	32.08	<0.01*	376	45.44	<0.01*	435	29.45	<0.01*	332	33.91	<0.01*
Require upper limb or hand force when working	641	7.59	<0.01*	442	7.17	<0.01*	429	13.92	<0.01*	520	16.42	<0.01*	382	10.09	<0.01*
Using vibration tools	323	31.26	<0.01*	245	47.39	<0.01*	239	52.44	<0.01*	279	49.47	<0.01*	215	47.90	<0.01*
Driving a vehicle	348	1.35	0.24	249	3.89	0.04*	241	5.10	0.02*	269	0.14	0.70	215	4.19	0.04*
Working in an uncomfortable posture	490	167.24	<0.01*	349	136.56	<0.01*	343	153.07	<0.01*	379	108.26	<0.01*	294	105.52	<0.01*
Doing the same job almost every day	671	28.14	<0.01*	461	20.33	<0.01*	436	14.01	<0.01*	533	22.57	<0.01*	392	14.57	<0.01*
Work content changes every day	119	0.97	0.32	93	5.40	0.02*	81	1.47	0.22	95	0.87	0.34	81	5.53	0.01*
Rotating job with colleagues	284	11.08	<0.01*	213	0.75	0.38	205	0.44	0.50	254	0.07	0.78	200	1.18	0.27
Finishing work in the same workshop	627	4.72	0.03*	427	1.98	0.15	409	2.57	0.10	508	11.16	<0.01*	378	11.13	<0.01*
Working outdoors	38	1.87	0.17	34	8.85	<0.01*	29	4.29	0.03*	33	3.33	0.06	33	13.86	<0.01*
Performing repetitive movements	541	64.91	<0.01*	379	53.71	<0.01*	361	50.02	<0.01*	463	104.94	<0.01*	335	62.88	<0.01*
Exposure to cold, cool breeze or temperature changes when working	234	61.32	<0.01*	174	62.28	<0.01*	164	55.42	<0.01*	202	74.50	<0.01*	161	77.00	<0.01*
Taking shift work	458	1.28	0.25	341	13.46	<0.01*	314	5.78	0.01*	411	30.29	<0.01*	302	20.41	<0.01*
Often working overtime	654	27.65	<0.01*	449	19.28	<0.01*	431	21.71	<0.01*	526	31.16	<0.01*	385	18.07	<0.01*
Adequate rest time	184	97.52	<0.01*	131	55.25	<0.01*	138	35.27	<0.01*	159	54.99	<0.01*	122	33.00	<0.01*
Starting working again after a break	648	11.05	<0.01*	439	3.77	0.05	420	4.27	0.03*	519	13.17	<0.01*	384	10.68	<0.01*
Ability to decide when to rest and work independently	128	0.00	0.97	103	3.62	0.05	93	1.38	0.23	98	0.19	0.66	95	7.22	<0.01*
Ability to decide when to take a break independently	130	0.11	0.74	92	0.41	0.52	85	0.07	0.79	93	0.82	0.36	79	0.41	0.52
Department staff shortage	284	62.25	<0.01*	191	35.49	<0.01*	186	38.62	<0.01*	233	58.79	<0.01*	180	54.77	<0.01*
Taking over another's shift frequently	140	49.79	<0.01*	118	80.28	<0.01*	100	46.40	<0.01*	120	54.91	<0.01*	108	87.52	<0.01*
Back bending forward when working	517	29.18	<0.01*	362	26.49	<0.01*	357	38.62	<0.01*	-	-	-	-	-	-
Turning around frequently	570	11.09	<0.01*	410	24.25	<0.01*	403	38.10	<0.01*	-	-	-	-	-	-
Trunk bending and twisting simultaneously	467	53.34	<0.01*	346	70.78	<0.01*	345	93.52	<0.01*	-	-	-	-	-	-
Repeating the same movement on the trunk	533	80.35	<0.01*	389	91.83	<0.01*	377	98.50	<0.01*	-	-	-	-	-	-

Keeping back in the same position for a long time	477	57.40	<0.01*	356	80.01	<0.01*	325	52.56	<0.01*	-	-	-	-	-	-
Bending over for a long time	290	93.79	<0.01*	227	118.61	<0.01*	223	128.24	<0.01*	-	-	-	-	-	-
Keep trunk twisting for a long time	324	79.12	<0.01*	260	123.36	<0.01*	243	105.62	<0.01*	-	-	-	-	-	-
Neck bending forward when working	603	26.68	<0.01*	397	5.10	0.02*	385	8.70	<0.01*	-	-	-	-	-	-
Keeping neck in the same position for a long time	496	119.03	<0.01*	335	68.97	<0.01*	301	38.94	<0.01*	-	-	-	-	-	-
Keeping head down for a long time when working	373	128.01	<0.01*	258	87.29	<0.01*	233	59.72	<0.01*	-	-	-	-	-	-
Neck twisting for a long time while work	309	129.26	<0.01*	241	152.45	<0.01*	205	85.25	<0.01*	-	-	-	-	-	-
Bending wrists up and down frequently when working	-	-	-	-	-	-	-	-	-	502	124.90	<0.01*	372	92.47	<0.01*
Bending wrist for a long time	-	-	-	-	-	-	-	-	-	416	211.19	<0.01*	305	145.24	<0.01*
Often placing wrists on the edge of hard and angular objects(e.g. a table edge)	-	-	-	-	-	-	-	-	-	425	198.59	<0.01*	307	125.41	<0.01*
Holding tools/objects in pinch grip when working	-	-	-	-	-	-	-	-	-	514	77.82	<0.01*	371	41.20	<0.01*
Working with hands below shoulders	-	-	-	-	-	-	-	-	-	450	0.03	0.85	326	0.87	0.35

Note. other staff included technical management personnel and auxiliary workers; -:not applicable for the body part; b: not presented, *n*≤5

Table S2. Univariate analyses for WMSDs in shoulders and lower extremities among male workers.

Factor	Shoulders			Hips/thighs			Knees			Ankles/feet		
	n	**χ2**	***P***	***n***	**χ2**	***P***	***n***	**χ2**	***P***	***n***	**χ2**	***P***
Age (years)		0.73	0.86		1.80	0.61		0.13	0.98		7.87	0.04*
<25	49			39			37			68		
25~	223			179			170			239		
35~	121			90			87			129		
45~	25			16			21			19		
BMI (Body mass index, kg/m^2)		4.66	0.19		4.63	0.20		4.62	0.20		4.76	0.19
<18.5	48			32			34			44		
18.5~	262			201			201			284		
24.0~	94			82			73			113		
28.0~	14			9			7			14		
Career length (years)		7.55	0.05		4.63	0.20		5.97	0.11		6.17	0.10
1~2	271			215			203			311		
3~5	114			84			81			113		
6~10	22			15			19			18		
10~	11			10			12			13		
Educational level[a]			0.09			0.06			0.10			0.03*
Junior high school and below	222			179			173			239		
High school or technical secondary school	158			121			120			177		
College or university	36			22			21			37		
Postgraduate degree and above	b			b			b			b		
Marital status		2.25	0.32		1.00	0.60		2.57	0.27		0.27	0.87
Never married	163			131			126			194		
Married	243			185			183			249		
Else (divorced or widowed)	12			8			6			12		
Monthly income[a]		5.10	0.16		6.56	0.08		3.72	0.29		3.01	0.38
≤1000RMB	7			6			b			6		
1001-3000RMB	8			b			b			6		
3001-5000RMB	154			126			114			165		
＞5000RMB	249			187			192			278		
Physical exercise		11.25	0.02*		6.05	0.19		1.22	0.87		7.36	0.11
Never	142			113			110			170		
sometimes	241			179			162			239		
2~3 times a month	14			14			16			18		
1~2 times a week	12			9			17			17		
>3 times a week	9			9			10			11		
Smoking habits[a]		1.87	0.17		0.09	0.76		0.83	0.36		1.21	0.27
Non-smokers	134			111			103			149		
Smokers	284			213			212			306		
Physical health status		91.76	<0.01*		90.67	<0.01*		74.83	<0.01*		79.87	<0.01*
Good	150			113			114			174		
Moderate	225			171			164			236		
Poor	37			31			30			36		
Very poor	6			9			7			9		
Dominant hand		0.00	0.97		0.08	0.76		2.75	0.09		0.02	0.86
Right	375			289			274			409		
Left	43			35			41			46		
Type of work		4.02	0.04*		4.94	0.02*		8.88	<0.01*		8.66	<0.01*
Frontline workers	381			298			294			420		
Other staff*	37			26			21			35		
Prolonged standing	400	3.27	0.07	310	2.41	0.12	299	0.88	0.34	442	10.79	<0.01*
Prolonged sitting	114	0.02	0.88	74	4.07	0.04*	88	0.02	0.87	107	4.41	0.03*
Prolonged squatting /kneeling	163	10.83	<0.01*	127	8.57	<0.01*	126	10.27	<0.01*	173	8.85	<0.01*
Carrying heavy loads (more than 20 kg each time)	346	13.87	<0.01*	278	20.82	<0.01*	274	25.08	<0.01*	381	19.65	<0.01*
Requiring upper limb or hand force when working	391	6.64	0.01*	307	9.00	<0.01*	296	6.05	0.01*	433	15.73	<0.01*
Using vibration tools	231	30.7	<0.01*	185	30.22	<0.01*	192	47.25	<0.01*	239	20.62	<0.01*
Driving a vehicle	256	5.55	0.01*	191	1.33	0.24	182	0.48	0.48	264	0.94	0.33
Working in an uncomfortable posture	303	93.67	<0.01*	238	76.33	<0.01*	231	73.16	<0.01*	328	99.73	<0.01*
Doing the same job almost every day	400	20.02	<0.01*	305	8.41	<0.01*	298	9.86	<0.01*	430	14.48	<0.01*
Work content changes every day	77	0.26	0.61	69	3.54	0.06	62	1.11	0.29	83	0.18	0.66
Rotating job with colleagues	189	0.88	0.34	140	2.49	0.11	140	1.18	0.27	196	3.92	0.04*
Finishing work in the same workshop	383	13.26	<0.01*	290	3.94	0.04*	286	7.02	<0.01*	413	10.60	<0.01*
Working outdoors	28	2.77	0.09	27	8.21	<0.01*	31	16.92	<0.01*	27	0.88	0.34
Performing repetitive movements	331	54.60	<0.01*	257	41.49	<0.01*	253	45.26	<0.01*	356	53.47	<0.01*

Exposure to cold, cool breeze or temperature changes when working	147	30.94	<0.01*	135	59.25	<0.01*	133	61.15	<0.01*	166	43.00	<0.01*
Taking shift work	295	8.94	<0.01*	232	8.97	<0.01*	223	6.92	<0.01*	324	11.90	<0.01*
Often working overtime	394	18.30	<0.01*	302	9.51	<0.01*	301	19.26	<0.01*	432	24.86	<0.01*
Adequate rest time	118	40.20	<0.01*	89	33.35	<0.01*	81	40.34	<0.01*	126	47.83	<0.01*
Starting working again after a break	387	8.59	<0.01*	302	8.44	<0.01*	288	3.30	0.06	418	6.57	0.01*
Ability to decide when to rest and work independently	88	1.03	0.30	70	1.31	0.25	71	2.47	0.11	84	0.20	0.64
Ability to decide when to take a break independently	94	2.08	0.14	72	1.25	0.26	72	1.96	0.16	95	0.33	0.56
Department staff shortage	183	35.33	<0.01*	160	55.41	<0.01*	164	70.77	<0.01*	207	50.47	<0.01*
Taking over another's shift frequently	86	21.77	<0.01*	81	42.43	<0.01*	79	41.68	<0<0.01*	92	21.74	<0.01*
Back bending forward when working	303	19.11	<0.01*	-	-	-	-	-	-	-	-	-
Turning around frequently	361	17.32	<0.01*	-	-	-	-	-	-	-	-	-
Trunk bending and twisting simultaneously	312	61.42	<0.01*	-	-	-	-	-	-	-	-	-
Repeating the same movement on the trunk	339	66.01	<0.01*	-	-	-	-	-	-	-	-	-
Keeping back in the same position for a long time	297	50.10	<0.01*	-	-	-	-	-	-	-	-	-
Bending over for a long time	186	75.04	<0.01*	-	-	-	-	-	-	-	-	-
Keeping trunk twisting for a long time	222	92.39	<0.01*	-	-	-	-	-	-	-	-	-
Neck bending forward when working	355	15.35	<0.01*	-	-	-	-	-	-	-	-	-
Keeping neck in the same position for a long time	274	52.92	<0.01*	-	-	-	-	-	-	-	-	-
Keeping head down for a long time when working	195	51.85	<0.01*	-	-	-	-	-	-	-	-	-
Neck twisting for a long time while work	186	83.43	<0.01*	-	-	-	-	-	-	-	-	-
Working with hands below shoulders	325	3.80	0.05	-	-	-	-	-	-	-	-	-
Stretching or changing leg posture frequently when working	-	-	-	272	10.08	<0.01*	260	6.23	.013*	393	26.98	<0.01*
Keeping knees bent for a long time when working	-	-	-	152	104.64	<0.01*	144	91.04	<0.01*	173	57.88	<0.01*
Lower extremities often repeating the same movement	-	-	-	230	95.76	<0.01*	221	86.86	<0.01*	300	91.07	<0.01*

Note. other staff include technical management personnel and auxiliary worker; -: not applicable for the body part; a: tested by Fisher's exact test; b: not presented, $n \leq 5$

Table S3. Univariate analyses for WMSDs in shoulders and lower extremities among female workers

Factor	**Shoulders**			**Hips/thighs**			**Knees**			Ankles/feet		
	n	χ^2	***P***	***n***	χ^2	***P***	***n***	χ^2	***P***	***n***	χ^2	***P***
Age (years)		7.39	0.06		1.11	0.77		1.49	0.68		1.78	0.61
<25	19			8			10			15		
25~	109			53			49			73		
35~	61			33			36			56		
45~	16			11			8			13		
BMI (Body mass index, kg/m2)		0.62	0.89		0.10	0.99		0.47	0.92		0.98	0.80
<18.5	30			17			16			25		
18.5~	142			71			69			104		
24.0~	28			14			15			23		
28.0~	b			b			b			b		
Career length (years)		4.00	0.26		0.72	0.86		1.34	0.71		2.18	0.53
1~2	140			79			77			114		
3~5	44			18			19			34		
6~10	12			6			6			b		
10~	9			b			b			b		
Educational level[a]			0.20			0.27			<0.01*			<0.01*
Junior high school and below	137			79			78			120		
High school or technical secondary school	48			22			24			35		
College or university	20			b			b			b		
Postgraduate degree and above	b			b			b			b		
Marital status		0.90	0.63		1.37	0.50		1.72	0.42		2.03	0.36
Never married	33			11			11			18		
Married	167			91			90			136		
Else (divorced or widowed)	b			b			b			b		
Monthly income[a]			0.62			0.15			0.87			0.31
≤1000RMB	b			b			b			b		
1001-3000RMB	6			b			b			b		
3001-5000RMB	120			63			60			87		
＞5000RMB	78			40			40			65		
Physical exercise		2.12	0.71		2.79	0.57		2.08	0.70		3.75	0.43
Never	90			45			43			76		
sometimes	96			50			53			70		
2~3 times a month	6			b			b			b		
1~2 times a week	11			6			b			b		
>3 times a week	b			b			b			b		
Smoking habits[a]			0.72			0.79			0.23			0.40
Non-smokers	203			104			103			154		
Smokers	b			b			b			b		
Physical health status		24.49	<0.01*		11.27	<0.01*		22.61	<0.01*		18.05	<0.01*
Good	84			43			39			67		
Moderate	104			55			52			76		
Poor	12			6			9			12		
Very poor	b			b			b			b		
Dominant hand		1.83	0.17		0.01	0.91		0.23	0.62		0.92	0.33
Right	197			99			98			145		
Left	8			6			b			12		
Type of work		0.86	0.35		3.25	0.07		5.37	0.02*		18.58	<0.01*
Frontline workers	171			96			96			152		
Other staff*	34			6			7			b		
Prolonged standing	175	0.90	0.34	94	0.47	0.48	96	3.46	0.06	150	10.87	.001*
Prolonged sitting	72	0.47	0.49	29	1.53	0.21	28	1.75	0.18	38	6.38	.012*
Prolonged squatting /kneeling	65	4.59	.032*	35	3.47	0.06	39	8.65	<0.01*	55	8.14	.004*
Carrying heavy loads (more than 20 kg each time)	120	5.87	.015*	69	10.19	<0.01*	73	18.10	<0.01*	103	15.75	<0.01*
Requiring upper limb or hand force when working	175	0.15	0.69	96	4.26	0.03*	96	6.56	0.01*	148	13.22	<0.01*
Using vibration tools	54	2.40	0.12	37	11.23	<0.01*	35	8.97	<0.01*	52	12.34	<0.01*
Driving a vehicle	59	0.14	0.70	35	1.82	0.17	35	2.22	0.13	48	0.74	0.38
Working in an uncomfortable posture	126	44.25	<0.01*	70	32.25	<0.01*	66	25.61	<0.01*	98	35.41	<0.01*
Doing the same job almost every day	200	9.47	.002*	101	2.40	0.12	100	3.57	0.05	153	6.64	0.01*
Work content changes every day	25	0.15	0.69	17	2.60	0.10	16	1.90	0.16	23	1.88	0.17
Rotating job with colleagues	81	1.64	0.2	49	0.44	0.50	50	1.13	0.28	67	0.05	0.80
Finishing work in the same workshop	183	0.00	0.94	94	0.00	0.96	92	0.00	0.97	144	1.01	0.31
Working outdoors	b	0.27	0.59	b		1.00	b		1.00	b		0.61
Performing repetitive movements	157	12.72	<0.01*	76	2.21	0.13	76	3.18	0.07	115	4.44	0.03*
Exposure to cold, cool breeze or temperature changes when working	58	22.55	<0.01*	31	12.92	<0.01*	33	18.22	<0.01*	46	19.55	<0.01*

Taking shift work	131	0.39	0.53	72	2.12	0.14	68	0.78	0.37	115	9.71	<0.01*
Often working overtime	186	5.28	.022*	97	4.29	0.03*	96	5.28	0.02*	149	12.67	<0.01*
Adequate rest time	65	16.82	<0.01*	32	9.40	<0.01*	29	12.46	<0.01*	43	21.84	<0.01*
Starting working again after a break	193	4.58	.032*	97	0.68	0.40	100	6.19	0.01*	148	3.55	0.05
Ability to decide when to rest and work independently	37	0.73	0.39	18	0.10	0.74	13	0.97	0.32	20	1.45	0.22
Ability to decide when to take a break independently	29	0.01	0.89	10	1.79	0.18	10	1.61	0.20	13	4.67	0.03*
Department staff shortage	52	2.81	0.09	31	5.01	0.02*	31	5.59	0.01*	43	4.41	0.03*
Taking over another's shift frequently	36	22.94	<0.01*	19	12.06	<0.01*	19	12.73	<0.01*	26	13.14	<0.01*
Back bending forward when working	165	13.91	<0.01*	-	-	-	-	-	-	-	-	-
Turning around frequently	154	1.87	0.17	-	-	-	-	-	-	-	-	-
Trunk bending and twisting simultaneously	113	6.67	.010*	-	-	-	-	-	-	-	-	-
Repeating the same movement on the trunk	144	22.48	<0.01*	-	-	-	-	-	-	-	-	-
Keeping back in the same position for a long time	138	15.16	<0.01*	-	-	-	-	-	-	-	-	-
Bending over for a long time	81	25.13	<0.01*	-	-	-	-	-	-	-	-	-
Keeping trunk twisting for a long time	84	13.74	<0.01*	-	-	-	-	-	-	-	-	-
Neck bending forward when working	183	9.47	.002*	-	-	-	-	-	-	-	-	-
Keeping neck in the same position for a long time	158	33.37	<0.01*	-	-	-	-	-	-	-	-	-
Keeping head down for a long time when working	120	26.70	<0.01*	-	-	-	-	-	-	-	-	-
Neck twisting for a long time while work	94	39.69	<0.01*	-	-	-	-	-	-	-	-	-
Working with hands below shoulders	164	0.26	0.61	-	-	-	-	-	-	-	-	-
Stretching or changing leg posture frequently when working	-	-	-	84	2.88	0.09	82	2.51	0.11	123	2.64	0.10
Keeping knees bent for a long time when working	-	-	-	30	5.22	0.02*	33	10.11	<0.01*	41	4.15	0.04*
Lower extremities often repeat the same movement	-	-	-	46	8.4	<0.01*	52	19.31	<0.01*	78	28.39	<0.01*

Note. other staff include technical management personnel and auxiliary workers; -: not applicable for the body part; a: tested by Fisher's exact test; b: not presented, $n \leq 5$
